# The interaction of linguistic and arithmetic factors affects adult performance on arithmetic word problems

**DOI:** 10.1007/s10339-019-00948-5

**Published:** 2020-01-22

**Authors:** Gabriella Daroczy, Detmar Meurers, Jürgen Heller, Magdalena Wolska, Hans-Christoph Nürk

**Affiliations:** 1 Department of Psychology, Eberhard Karls University Tuebingen, Tübingen, Germany; 2 Department of Linguistics, Eberhard Karls University Tuebingen, Tübingen, Germany; 3 LEAD Research Network, Eberhard Karls University Tuebingen, Tübingen, Germany

**Keywords:** Arithmetic word problem, Nominalization, Carry effect, Lexical consistency, Addition, Subtraction, Linguistic complexity

## Abstract

Performance on word problems is influenced by linguistic and arithmetic factors, and by their interaction. To study these factors and interactions, we manipulated linguistic and arithmetic factors independently in a within-participant design that included complexity parameters (a) in the domain of arithmetic: carry/borrow (no-carry/borrow vs. carry/borrow), operation (addition vs. subtraction), (b) in the domain of linguistics: nominalization (nominalized vs. verbalized form), and (c) linking the two domains: lexical consistency (linguistic predicate locally consistent vs. inconsistent with mathematical operation). Response times of 25 students solving 320 one-step word problems were measured. All four factors showed a main effect on response times, and interactions between linguistic and arithmetic factors affected response times. These interactions were observed when the linguistic and arithmetic factors were conceptually linked. Our results highlight that not only the linguistic and arithmetic complexities of an item contribute to the difficulty of a word problem, but linguistic and arithmetic factors interact. We discuss the theoretical implications for the numerical and the linguistic domain as well as the possible impact of domain-general characteristics, such as working memory limitations as a potential reason for the observed interactions between numerical and linguistic attributes.

**Electronic supplementary material:**

The online version of this article (10.1007/s10339-019-00948-5) contains supplementary material, which is available to authorized users.

## Introduction

Word problems, where a mathematical problem is presented using language, play an important role in the school curriculum, and they have been shown to be difficult for people of all ages (Boonen et al. 2013; Hegarty et al. 1992; Lewis and Mayer 1987; Nesher and Teubal 1975; Riley 1984; Verschaffelet al. 1992). A wide range of factors, from individual traits to the socioeconomic background, the school environment, and the scoring system influence performance on word problems. Here we focus on the fact that the ability to successfully solve a word problem depends on task characteristics: how the text is formulated (i.e., linguistic features), and how difficult the arithmetic operations are (i.e., arithmetic features). The connection between arithmetic and linguistic factors is not yet fully understood. One reason is that arithmetic and linguistic factors are confounded in many studies, making it hard to draw conclusions about which of the factors—i.e., the arithmetic or the linguistic one—are responsible for increased response times or higher error rates. For example, experiments with word problems often differ in the number of computational steps: Many studies compare performance on one-step problems, involving one calculation, to two-step word problems, which require two calculations involving multiplication or division in combination with addition or subtraction. Two-step problems are usually arithmetically and linguistically more complex, containing longer sentences with more propositions. Generally, they also are more complex in terms of domain-general factors given that for two-step problems more information must be kept in working memory (Fuchs et al. 2016). Thus, when the additional step in two-step problems impacts the speed and/or the accuracy of solving the task (Muth 1992; Quintero 1983), it is not clear whether the increased difficulty is due to arithmetic complexity, linguistic complexity, or the domain-general requirement of more cognitive resources.

In this study we spell out and investigate a distinction, which has not been prominent so far, between (1) linguistic and numerical factors that are conceptually independent of one another, in the sense that they can also be manipulated independently, and (2) those which are intrinsically linked. Indeed, some linguistic and arithmetic factors are not separable. For example, a keyword that provides a hint for the solution of a word problem is a linguistic factor that can only be chosen based on the mathematical operation necessary to solve the text problem (e.g., Van der Schoot et al. 2009). Similarly, the arithmetic factor operation is directly connected to the lexical choice as a linguistic feature of word problems (Lave 1992), as the concepts of addition and subtraction are commonly expressed by “giving” and “taking” in everyday language (Carraher et al. 1988). On the other hand, there are independent linguistic and arithmetic factors, where the other domain remains unaffected. For example, changing the name of the protagonist in a word problem (Abedi and Lord 2001) does not generally change the underlying mathematics. Similarly, whether the numbers in a word problem are two-digit or three-digit numbers (Thevenot and Oakhill 2006) does not change the language. Such intrinsically related and unrelated factors may affect the process of solving a word problem to different extents. At the same time, linguistic complexity and arithmetic complexity in word problems are often seen as subsequent additive processes, which do not interact with each other. If such a theoretical assumption were true, then one could argue that it is enough to study arithmetic complexity in common (nonverbal) arithmetic tasks. However, studying arithmetic complexity in isolation would not make it possible to observe and distinguish different sources of variance in solving word problems and establish whether they arise from independent linguistic factors, independent arithmetic factors, or factors involving both domains. It is also impossible to pose the general question as to whether all of these factors may arise from domain-general requirements for cognitive resources. Therefore, in our view it is necessary to explore the manipulation of both linguistic and arithmetic factors in a word problem study independently.

In the sections Linguistic Complexity and Arithmetic Complexity, we will introduce the related and unrelated factors. Following that, in the section Underlying Cognitive Processes of Arithmetic Problem Solving, we give an overview of existing models for solving word problems. Finally, in the section Interaction of Linguistic and Arithmetic Factors, we will first elaborate on how the presence or absence of an interaction between the factors could support the existing models, before closing with a discussion of potential interactions between unrelated and related arithmetic and lexical complexity.

### Linguistic complexity

Performance on one-step problems is strongly dependent on the wording of the problem (De Corte 1988; De Corte et al. 1985; Vicente et al. 2007). Some linguistic factors affect the underlying mathematical structure, while other factors do not. Factors not affecting the underlying mathematical structure include general descriptive characteristics (e.g., overall number of words, average sentence length), grammatical features, and most lexical properties (Haag et al. 2013; Richards and Schmidt 2013). Factors that affect the mathematical structure, like the consistency of the key word expressing the operation (Domahs et al. 2006; Pape 2003; Van der Schoot et al. 2009; Verschaffel et al. 1992), contribute to the difficulty of word problems. The relation of these factors to the problem solving process is often not clear.

#### Linguistic factors unrelated to arithmetic factors

To begin with, in some cases modification of the language of a word problem without changing the semantic and mathematical structure leads to higher success rates (Cummins et al. 1988; Davis-Dorsey et al. 1991; Stern and Lehrndorfer 1992; Vicente et al. 2007). However, it is important to note that richer text can also bring about additional difficulty, depending on the linguistic complexity of the added text. Adding non-relevant information (Barbu and Beal 2010), a modification that does not change the mathematical structure of the text, can lower success rates. Nevertheless, many such text manipulations unrelated to arithmetic factors are reported to improve solution accuracy:

1. The text is described more richly to clarify the situation (Stern and Lehrndorfer 1992). Consider the example from Hudson (1983): without situational rewording “Here are some birds and here are some worms. How many more birds than worms are there?’ and with situational rewording “Here are some birds and here are some worms. Suppose the birds all race over and each one tries to get a worm. How many birds won’t get a worm?”
2. Conceptual rewording where the underlying structure is highlighted through simple language modification (e.g., Vicente et al. 2008).
3. Introduction of personalization (Abedi and Lord 2001; Reusser 1988) to the context.

It is also suggested that uncommon vocabulary in mathematical tasks is negatively related to performance (Abedi and Lord 2001; Shaftel et al. 2006). However, other studies such as that of Bergqvist et al. (2012) did not find any evidence for this relation.

In choosing one linguistic factor that does not change the underlying mathematics but increases the complexity of the language, we decided on *nominalization*. *Nominalization* is the process of turning verbs, often expressing actions, into nouns (Francis 1989), thus increasing the number of noun phrases in the text. For instance, the verb *“to earn” can be nominalized into “the earning,”* as illustrated by the following word problem examples, with the verbal form in (1) and the nominal form in (2) shown in bold (in German, the language we are focusing on in the experiments, nominalized forms are systematically used and typical for academic language, while in English such nominalization can be marginal):

1. A man saved some money. He had 82 euros.
  The next day, he **earns** 15 euros.
  How much money does the man have now?
2. A man saved some money. He had 82 euros.
  The next day, he is happy about the **earning** of 15 euros.
  How much money does the man have now?

These examples differ in terms of their linguistic complexity, but both problems can be solved with the same arithmetic operation, in this case addition. It is known that nominalization increases the difficulty of comprehension (Halliday et al. 2014; To et al. 2013) independent of the domain of word problems. The number of noun phrases has thus been referred to as the foremost predictor of text difficulty (Haag et al. 2013). In addition, nominal style is characteristic of academic language, in legal, political, or scientific texts (Baratta 2010). It is also common in mathematical discourse (Perry et al. 1999). Nevertheless, nominalization has rarely been investigated in word problems. In one study that investigated nominalization, it was shown that if word problems contain a high density of nominalization, this significantly affects the solution rate for tenth grade students (Schlager et al. 2017). However, in the study, multi-step and more complex problems were used, manipulating various other linguistic features at the same time. While nominalization does not change the underlying mathematical structure and does not introduce additional arithmetic complexity into identifying the required calculation, we expect that nominalization, as a characteristic of complex language, should affect word problem performance.

#### Linguistic factors related to arithmetic factors

Complementing the unrelated linguistic factors just discussed, there also are linguistic factors that are related to the arithmetic underlying a word problem. The most prominent of such factors is *lexical consistency,* introduced in the work of Lewis and Mayer (1987). Lexical consistency concerns specific keywords in the text, the so-called cue words, which signal or hint toward particular arithmetic operations like addition or subtraction (Hinsley et al. 1977). A word problem is considered lexically consistent if the semantics of the cue words signal an operation that is congruent with the operation required for the correct solution. It can be illustrated by the following example:

(3) A man saves money on some purchases. He had 82 euros. He **earns** 15 euros. How much money does the man have now?

Here the cue word *earns* is associated with getting more, i.e., it infers the operation can be associated, in this case, with addition, and the solution indeed requires addition: 82 + 15. In contrast, in inconsistent word problems, the relational term hints at some other operation, usually the opposite.

(4) “A man saves money on some purchases. He **spent** 74 euros. He has 23 euros now. How much money did the man have?”

In example (4) the cue word *spent* is commonly associated with having less Euros than before; thus, it is associated with subtraction. However, in this example, computing the solution requires addition: 74 + 23 = 97. Such problems are called lexically inconsistent because the operation most frequently associated with the lexical meaning of the cue words is inconsistent with the operation needed for the correct solution in this particular context. Indeed, most errors in lexically inconsistent conditions are due to an erroneous choice of operation (Hegarty et al. 1992; Zawaiza and Gerber 1993). In this manuscript, we refer to lexical consistency as a linguistic factor *related* to arithmetic factors, because the word chosen is linked to the mathematical operation. Note, however, that it can be manipulated in a 2 × 2 design (lexical consistency × operation) since the operation to be carried out ultimately depends not on the most common context of use of a particular trigger word, but on the particular question asked by the given word problem. Lexically consistent and inconsistent problems can be constructed for both addition and subtraction, by combining the appropriate cue words for the operations with different question contexts. In the current study, we have done so.

The effect of lexical consistency on both solution time and accuracy has been studied previously (e.g., Hegarty et al. 1992; Hegarty et al. 1995; Pape 2003; Verschaffel et al. 1992). Compared to lexically consistent problems, lexically inconsistent ones are usually associated with increased solution time and lower solution accuracy, although some studies failed to detect effects. For example, Verschaffel et al. (1992) found a consistency effect for children but not for adults when they were presented one-step arithmetic comparison problems. The studies of Hegarty et al. (1992, 1995) detected the consistency effect in two-step arithmetic problems and showed that even students who successfully solve word problems need more time to derive solutions in the inconsistent condition. In line with this, we expect lexical consistency to affect word problem performance.

### Arithmetic complexity

Naturally, the complexity of a word problem also depends on arithmetic factors. For instance, choosing the correct operation strongly depends on the nature of the numbers in the problem (De Corte et al. 1990), and more difficult calculations might lead to lower solution accuracy (Thevenot and Oakhill 2006). For example, participants may show more calculation failures when operations requiring a carry are involved. The arithmetic factors can be grouped according to their relatedness to linguistic factors.

#### Arithmetic factors unrelated to linguistic factors

It is well known from the literature that arithmetic complexity in the form of basic number properties (e.g., Ashcraft 1992; Nuerk et al. 2011) and the complexity of underlying arithmetic computations (e.g., Göbel et al. 2014) play a major role in common arithmetic problems. Surprisingly, there has been relatively little effort to investigate such basic forms of arithmetic complexity in word problems, although it is known that some errors originate from arithmetic computation errors themselves (Kingsdorf and Krawec 2014; Raduan 2010). Multiple effects of numerical cognition are known to contribute to the difficulty of an arithmetic problem (see, Nuerk et al. (2011) for multi-digit numbers). We decided to start with arguably the most ubiquitous effect of multi-digit addition complexity in arithmetic cognition, the carry and borrow effect. The difficulty of two-digit addition and subtraction increases whenever a carry or borrow operation is required (Artemenko et al. 2018). In carry problems, a 1 needs to be carried from the unit slot to the tenths slot. An example for arithmetic calculation with carry is 14 + 39 = 53, where 4 + 9 = 13 and the digit 1 is the carry; an arithmetic calculation with no-carry is illustrated by 11 + 12 = 23. Similarly, the borrow operation in subtraction is needed whenever the unit of the minuend is smaller than the unit of the subtrahend so that a decade must be borrowed from the minuend. Example for arithmetic calculation with borrow is 34 − 19 = 15 where 4 − 9 = −5 so we compute (10 − 9) + 4 = 5 and the 10 is obtained by taking (“borrowing”) 1 from the next digit to the left. In contrast, 35 − 12 = 23 is an example for an arithmetic calculation with non-borrow. In sum, carrying and borrowing increases the arithmetic complexity of a problem. Importantly, the carry or borrow operation is unrelated to any linguistic property. Merely the numbers are changed, the problem is otherwise identical. It has been shown that in carry/borrow conditions compared to non-carry/non-borrow conditions, the response time increases for two-digit addition in children and adults (Artemenko et al. 2018).

In sum, there are reliable evidence outside of the word problem literature that the carry effect influences the arithmetic performance of both children and adults and evidence that other arithmetic effects also play a role in word problems. Therefore, we hypothesize that the carry effect should also influence performance on word problems, but this has—to the best of our knowledge—never been systematically tested.

#### Arithmetic factors related to linguistic factors

One of the major challenges in a word problem is to find the correct arithmetic operation after understanding the text. Nevertheless, even after the correct operation is successfully detected, the operations might still vary in their difficulty. Most word problem research addresses addition and/or subtraction as the operation (e.g., Carpenter et al. 1981; De Corte and Verschaffel 1987; Fennema et al. 1996; García et al. 2006; Klein et al. 2009; Moeller et al. 2011). Presumably, this is because the limits of mental arithmetic are quickly reached in more complex operations, such as multiplication or division (Swanson 2004).

In our experiments, we also focus the word problem operation tasks on addition and subtraction. Incorporating these two operations is necessary. If the same operation needs to be computed for all problems, e.g., addition, people will not try to read and understand the text anymore, but simply add the numbers found in the text, no matter what the text says.

With regard to the effect of the operation itself, it was shown that subtraction is generally more difficult (Artemenko et al. 2015) and elicits greater response times (Orrantia et al. 2012) than addition. Additionally, many types of subtraction tasks can be solved by various strategies, including the indirect addition or subtraction by addition strategy (De Corte and Verschaffel 1987; Torbeyns et al. 2009). Therefore, we expect that subtraction should increase the time needed to solve a word problem, as compared to addition.

Concluding, we sketched arguments for why each of the above-mentioned linguistic and arithmetic factors could affect performance on word problems. In order to differentiate the extent to which they influence performance and to explore their interactions, we therefore divided task characteristics into unrelated and related factors. Additionally, so far there is very little information about the relationship of these factors to problem solving processes. Differentiating the related and unrelated factors may also be important because different word problem solving models would suggest different interactions between those factors—which we discuss in the following section.

### Underlying cognitive processes of arithmetic problem solving

#### Models of problem solving

Several studies on word problems investigated the underlying cognitive processing (De Corte et al. 1990; Hegarty et al. 1992, 1995; Verschaffel et al. 1992). It is hypothesized that solving word problems requires four distinct phases (Mayer 1984): an initial reading phase (i.e., translation of the text), integration (i.e., mental representation and the construction of the problem model), planning (i.e., generating a solution plan), and solution execution (i.e., calculation). However, a comprehensive theory of problem solving is still lacking (Passolunghi and Pazzaglia 2004). The existing models for solving word problems differ in several respects, such as in the nature and the origin of the internal problem model—the mathematization of the text—and whether the problem solving phases are fully separable or not. First, it is under debate whether the internal problem representation is a result of schemas, a situated model or a mental representation model. According to Kintsch and Greeno (1985), the problem model relies only on schemas stored in long-term memory and the problem model is constructed from the text base and relies on the problem-solver’s previous knowledge as well as the text. Van Dijk and Kintsch (1983) extended this prior schema-based problem model construction with a situation model, which corresponds to a level of representation that specifies the agents, actions, and relationships between events in everyday contexts. Finally, some argue that ad hoc transient mental representations are constructed for each problem encountered with the help of working memory (e.g., Thevenot and Barrouillet 2015; Thevenot et al. 2007). Finally, the problem solving models in the literature do not agree on whether the process of reading is completely separable from the process of solving. Studying the presence or absence of an interaction between related and unrelated factors from the linguistic and arithmetic domain could provide relevant evidence for this debate.

#### Interactions between related and unrelated factors and the models of problem solving

In the first model, the propositional theory, the text formulation determines the difficulty of constructing an adequate representation (Abedi and Lord 2001; Cummins et al. 1988; De Corte et al. 1985). This is supported by the documented strong connection between text comprehension and the solving of word problems (Boonen et al. 2013; Boonen et al. 2014; Kintsch and Greeno 1985). This model sees the phases of problem solving as clearly separable and serial. For example, in the case of an arithmetic word problem, the complexity of the text rather than the mathematical operations involved influences the processing of the problem (Nesher 1976). This is supported by Rabinowitz and Wooley (1995), who found no significant interaction between problem size (one-digit vs. two-digit numbers) and various problem types. According to the propositional model, the number difficulty (i.e., carry) should mainly affect the calculation/execution phase, and text difficulty (i.e., nominalization) mainly the initial text comprehension phase, and not the building of the problem model phase. Contrary to this, factors that are related to other arithmetic/linguistic factors should affect not only the first reading (in the case of lexical consistency, which has been shown to manifest itself in the second phase of problem solving) and the calculation (i.e., in the case of operation), but also the phase where the problem model is built. This is also supported by the fact that an interaction between operation and lexical consistency is often found in studies (e.g., Van der Schoot et al. 2009; Verschaffel et al. 1992).

On the other hand, other models suggest that the text formulation affects not only the first reading but also other phases in the solution process, and that problem comprehension and computational processes interact. Such an interaction could cause the computational requirements of the problem to interfere with problem representation. For instance, Thevenot and Oakhill (2005) suggested that the mental representation constructed when solving a word problem involves a re-enactment of the solvers’ experiences with the processing of magnitude information for quantities. The study of Munez et al. (2013) also suggests that in solving an arithmetic word problem, solvers construct a magnitude-based mental representation that goes beyond a conceptual representation in the form of propositions. This would suggest that number processing might also influence another stage of problem solving. This hypothesis is supported by De Corte et al. (1990), who questioned the sequential and linear character of a theoretical model of competent problem solving, especially with respect to more complex problem types. The theoretical model proposed by Bergqvist and Österholm (2010) also indicates that the process of solving word problems comprises a cyclical component revisiting the mental representation. This would also mean that reading interacts with other phases of problem solving. These non-sequential models would suggest an interaction, for example, between the unrelated arithmetic factors/linguistic factors and the other factors. This hypothesis is supported, for example, by the findings of Hegarty et al. (1992) or Verschaffel et al. (1992), who found that in word problems with linguistically marked words (“less than”) more time is needed to solve the problem compared to word problems with unmarked words (“more than”). Markedness suggests that in most languages, there is usually a complementary pair of adjectives, with one adjective being the ground (unmarked) form and the other being the derived opposite (marked) form. Which adjective is marked or not can be determined in three ways: The easiest and most consistent way is formal markedness. In this case, the form of the marked adjective is explicitly marked by a negating prefix, for example the prefix “in” turning “efficient” into “inefficient,” or the prefix “dis” that can mark “organized” to obtain “disorganized” (Zimmer et al. 1964). Two other ways to define which is the marked term are semantic and distributive markedness (Lyons 1977). An example of semantic markedness is “old” vs. “young,” where in neutral context the unmarked term (“old”) is the one used (“How old is the baby?”). Distributive markedness, on the other hand, is related to word frequency.

In conclusion, each model predicts different interactions. According to the propositional theory, we should find no interaction between related linguistic factors/arithmetic factors and unrelated arithmetic factors/linguistic factors given that this theory sees the initial reading, the calculation phase, and the building of the problem model phase as distinct, non-overlapping stages of problem solving. Applying the logic of Sternberg (1969), interactions are not possible because there is no common stage of processing for the cognitive processes underlying the manipulated factors. In contrast to propositional models, we should find an interaction between related and unrelated factors according to the models that consider problem solving to be sequential and cyclic because reading and number processing might both influence the problem model phase, i.e., the manipulated factors operate partially on a common stage of processing. In our case, we can associate the unrelated mathematical factor carry mostly with the calculation phase as it increases the difficulty of the calculation. On the contrary, the unrelated factor nominalization should be associated with the initial reading phase, because it increases the reading demand only. The factors lexical consistency and operation could be associated with the phases where the mental representation is constructed, as they are related to both linguistic and mathematical domains. This assumption is consistent with the literature suggesting both factors affect the second stage of problem solving (e.g., Hegarty et al. 1992). This indicates that an interaction between the unrelated carry factor and any other factors would suggest that the calculation process interacts with other problem solving phases. Similarly, an interaction between the nominalization factor and any other factors would suggest that the reading comprehension phrase interacts with other problem solving phases.

Nevertheless, models for solving word problems usually do not consider the joint investigation of numerical and textual difficulty, and their involvement in mental problem solving. For example, the role of numerical information is especially unclear and often not covered in the models. Therefore, in the next section, we will elaborate on the possible interactions of related and unrelated linguistic and mathematical task characteristics.

### Interaction of linguistic and arithmetic factors

Linguistic and arithmetic factors may influence the problem solving processes differentially. For instance, a focus on specific parts of word problems can be associated with certain problem solving strategies. Expressed in terms of the strategies characterized by Hegarty et al. (1992), students using a so-called direct translation strategy, where students select keywords and numbers from the text to carry out a computation on this shallow basis, would focus on a few words only, whereas students using a problem model strategy would pay more attention to the a broader range of words in the problem and build a situation model. However, under semantically less demanding conditions problem solvers can apply successful strategies (Van der Schoot et al. 2009). In addition, looking at the keywords without an understanding of the problem situation does not necessarily lead to a superficial solution process. As the difficulty of the text increases, the creation of the situation model and analytic processes operating on the selected items to generate inferences (Evans 1984) get more and more important.

According to the model of Daroczy et al. (2015), word problem difficulty comprises linguistic factors and arithmetic factors and these affect individual performance both directly and through mediator variables, such as domain-general attributes and solution strategies. Due to the joint resources, interactions with linguistic factors are to be expected and task characteristics should not affect the solution phases to the same extent. The interaction of linguistic and arithmetic factors should be present in the response time because some factors are not separable from the other domain and play an extensive role in building mental models, and we hypothesize that these factors should not affect the solution phases to the same extent. If the factors are related by their very definition there must be a direct connection for such related linguistic and cognitive factors given that the relation does not only appear when working memory or other domain-general resources are relevant. For example, subtraction is more heavily dependent on mental representation than a simple numerical manipulation, because different semantic classes correspond to the different types of conceptual knowledge needed to solve problems, such as knowledge about increases or decreases in quantity. The interaction with the related factor (lexical consistency, operation) should therefore be especially pronounced because they all overlap in the reading, comprehension, and execution phases. We expect this interaction to be over-additive, i.e., when the relation between operation and lexical consistency is such that both factors are difficult (lexically inconsistent subtraction problems)—due to limited resources—and we hypothesize longer response times than expected based on the main effects only.

However, according to the model of Daroczy et al. (2015), we would expect interactions not only between related factors but also between unrelated and other factors. The complex linguistic and mathematical task characteristics make the task more difficult (leading to main effects in appropriate designs), but they can increase domain-general attributes such as cognitive load because they impose on limited domain-general resources (Sweller 1994), and because they might share the same processing stage (e.g., Sternberg 1969). However, the mechanisms of and relationship to other domain-specific and domain-general factors are still under debate (Lee et al. 2009; Tolar et al. 2012; Zheng et al. 2011). Several studies have shown that both domain-general factors, such as working memory (Adams and Hitch 1997; Passolunghi and Siegel 2001; Swanson 2004), reading comprehension (Swanson and Beebe-Frankenberger 2004), and processing speed (Kail and Hall 1994) as well as domain-specific factors, such as arithmetic computing or concept formation (Fuchs et al. 2016), are related to word problem performance. Furthermore, for other domain-specific and domain-general factors, working memory was shown to play a role not only for children (e.g., the verbal and spatial components of working memory (Soltanlou et al. 2015)), but also for adults. For instance, for adults, the central executive is consistently found to be important for the carry operation in multi-digit arithmetic, e.g., Imbo et al. (2007), and in the domain of word problems working memory capacity influences the choice of solution strategies (Thevenot and Oakhill 2006). Working memory also plays a role in language acquisition and understanding prepositions (Ellis 1996). Its limitations affect the ability of elderly adults to process complex syntactic constructions (Norman et al. 1992). This means, for example, for factors unrelated to arithmetic/linguistic, such as carry and nominalization, we hypothesize that the interactions should still be observed but less consistently. We wish to note that such an interaction does not need to rely on a direct relationship between linguistic and arithmetic factors per se, for unrelated factors like nominalization an interaction may simply entail linguistic and arithmetic factors using the same type of domain-general resource at some stage of the solution process.

### Objectives

In this paper, we examine the role of linguistic and arithmetic factors in word problem solving performance. In designing the items, we manipulated linguistic complexity independent and orthogonal to the arithmetic complexity of the arithmetic problem underlying the word problem. We then designed the study such that for both linguistic and arithmetic complexity, there was one factor relating arithmetic and linguistic complexity and one unrelated factor. In particular, the linguistic factor lexical consistency relates to arithmetic complexity (namely operation), whereas the linguistic factor nominalization does not. Analogously, the arithmetic factor operation is related to linguistic complexity (namely lexical consistency), whereas the arithmetic factor carry/borrowing is not. Based on the literature reviewed above, we formulated the following hypotheses:

1. We hypothesize that all arithmetic and linguistic factors will show a main effect on performance (H1), which we have split up for each factor as follows:
  Regarding arithmetic, subtraction tasks should take significantly longer than word problems with addition (H1.1).
  Because of the carry effect, word problems with a carry operation will result in significantly longer response times (H1.2).
  The consistency effect will cause significantly longer response times for lexically inconsistent items (H1.3).
  Additionally, we assume that the nominal form is more difficult to understand even for adults and will increase the response time significantly (H1.4).
2. Interactions between arithmetic and linguistic factors are expected (H2):
  For linguistic/arithmetic factors which are related to other arithmetic/linguistic factors, such interactions should be particularly pronounced and more consistent, i.e., in the case of lexical consistency and operation (H2.1).
  For factors affecting only linguistic or arithmetic, i.e., carry and nominalization, the interactions should still be observed, but less consistently, i.e., in some cases they will be absent. (H2.2).

## Methods

### Participants

A total of 29 students participated in the experiment. All were native German speakers between 18 and 45 years old with normal or corrected (only with soft contact lenses) to normal vision. Neurological or psychological disorders were exclusion criteria. Four persons were excluded from the analysis for the following reasons: In two cases, the experiment had to be stopped due to technical difficulties. The other two subjects had an error rates that were too high. (Participants with an accuracy below 75% were removed.) Twenty-one of the remaining 25 persons (*M* = 22.08, SD = 2.59) were female (*M* = 21.86, SD = 2.23) and four were male (*M* = 23.25, SD = 3.77). For the remaining participants, the error range was from 1.0 to 13.3% (*M* = 7.0%; SD = 3.4%).

Participation was on a voluntary basis and was rewarded with either three subject hours toward course credits or with 20 Euros. Informed consent was given by all participants. The study was performed in accordance with the ethical standards of the Declaration of Helsinki.

### Stimuli and design

The study was a 2 × 2 × 2 × 2 design with the main factors: operation (addition/subtraction), carry (carry/non-carry), lexical consistency (consistent/inconsistent form), and nominalization. 320 simple arithmetic, one-step word problems in German were designed for the study. There were 16 conditions that consisted of 20 sentences (see Tables 1, 2). The four factors were manipulated simultaneously and orthogonally. Specifically, to explore the effect of arithmetic complexity, the numbers and operations were manipulated, while the text remained largely identical. Likewise, for exploring linguistic complexity, linguistic factors were manipulated, while the complexity of the arithmetic problem was kept constant. All sentences belonged to the type “Change”—namely Change 1, Change 2, Change 5, and Change 6—according to the categorization of Riley (1984). Change problems refer to dynamic situations in which some event changes the value of a quantity (Verschaffel and De Corte 1993). All four types of these mentioned problems were equally distributed. In half of the stimuli, the result set (160 tasks) was unknown, from which half belonged to the category Change 1 (80 tasks, addition), the other half to the category Change 2 (80 tasks, subtraction). From the other 160 problems—where the start set was unknown—half belonged to the category Change 5 (80 tasks, addition), and the other half Change 6 (80 tasks, subtraction). Change 1 and Change 2 are considered to be easier tasks than the more difficult Change 5 and Change 6 (Riley 1984) which means that both addition and subtraction tasks contain one easier and one more difficult type.

**Table 1:** 16 conditions

	Consistent form		Inconsistent form	
	Nominal form	Verbal form	Nominal form	Verbal form
Subtraction				
Carry/borrow	C1	C2	C3	C4
Non-carry/non-borrow	C5	C6	C7	C8
Addition				
Carry/borrow	C9	C10	C11	C12
Non-carry/non-borrow	C13	C14	C15	C16

**Table 2:** Examples for the 16 conditions

Condition	Sentences
C1	Ein Mann spart Geld für einige Anschaffungen. *A man saves money on some purchases.* Er bedauert *das Ausgeben* von 14 Euro. *He regrets****the spending****of 14 euros. (Glossing)* *He regrets spending 14 euros. (Translation)* Er hatte 61 Euro gehabt. Wie viel Geld hat er am Ende? *He had 61 euros. How much money does he have in the end*?
C2	Ein Mann spart Geld für einige Anschaffungen. *A man saves money on some purchases.* Er hat 18 Euro ausgegeben. *He spent 18 euros.* Er hatte 41 Euro gehabt. *He had 41 euros.* Wie viel Geld hat er am Ende? *How much money does he have in the end?*
C3	Ein Mann spart Geld für einige Anschaffungen. *A man saves money on some purchases.* Er hat jetzt 56 Euro. *He has 56 euros now.* Er hat sich über *das Verdienen* von 28 Euro gefreut. *He was about****the earning****of 28 euros happy. (Glossing)* He was happy to earn 28 euros. *(Translation)* Wie viel Geld hatte er am Anfang? *How much money did he have in the beginning?*
C4	Ein Mann spart Geld für einige Anschaffungen. *A man saves money on some purchases.* Er hat 36 Euro verdient. *He earned 36 euros.* Er hat jetzt 52 Euro. *He has 52 euros now.* Wie viel Geld hatte er am Anfang? *How much money did he have in the beginning?*
C5	Ein Mann spart Geld für einige Anschaffungen. *A man saves money on some purchases.* Er bedauert **das Ausgeben** von 12 Euro. *He regrets****the spending****of 12 euros. (Glossing)* *He regrets spending 12 euros. (Translation)* Er hatte 64 Euro gehabt. *He had 64 euros.* Wie viel Geld hat er am Ende? *How much money does he have in the end?*
C6	Ein Mann spart Geld für einige Anschaffungen. *A man saves money on some purchases.* Er hat 56 Euro. *He has 56 euros.* Er hat 31 Euro ausgegeben. *He spent 31 euros.* Wie viel Geld hat er am Ende? *How much money does he have in the end?*
C7	Ein Mann spart Geld für einige Anschaffungen. *A man saves money on some purchases.* Er hat jetzt 58 Euro. *He has 58 euros now.* Er hat sich über **das Verdienen** von 37 Euro gefreut. *He was about****the earning****of 37 euros happy. (Glossing)* *He was happy about earning 37 euros. (Translation)* Wie viel Geld hatte er am Anfang? *How much money did he have in the beginning?*
C8	Ein Mann spart Geld für einige Anschaffungen. *A man saves money on some purchases.* Er hat jetzt 76 Euro. *He has 76 euros now.* Er hatte 13 Euro verdient. *He had earned 13 euros.* Wie viel Geld hatte er am Anfang? *How much money did he have in the beginning?*
C9	Ein Mann spart Geld für einige Anschaffungen. *A man saves money on some purchases.* Er hatte 17 Euro. *He had 17 euros.* Er freut sich über **das Verdienen** von 58 Euro. *He is happy about****the earning****of 58 euros. (Glossing)* *He is happy about earning 58 euros. (Translation)* Wie viel Geld hat der Mann jetzt? *How much money does the man have now?*
C10	Ein Mann spart Geld für einige Anschaffungen. *A man saves money on some purchases.* Er hatte 34 Euro. *He had 34 euros.* Er verdient 49 Euro. *He earns 49 euros.* Wie viel Geld hat der Mann jetzt? *How much money does the man have now?*
C11	Ein Mann spart Geld für einige Anschaffungen. *A man saves money on some purchases.* Er bedauert **das Ausgeben** von 65 Euro. *He regrets****the spending****of 65 euros. (glossing)* *He regrets spending 65 euros. (translation)* Er hat jetzt 18 Euro. *He has 18 euros now.* Wie viel Geld hatte der Mann? *How much money did the man have?*
C12	Ein Mann spart Geld für einige Anschaffungen. *A man saves money on some purchases.* Er hat 19 Euro ausgegeben. *He spent 19 euros.* Er hat jetzt 32 Euro. *He has 32 euros now.* Wie viel Geld hatte der Mann? *How much money did the man have?*
C13	Ein Mann spart Geld für einige Anschaffungen. *A man saves money on some purchases.* Er hatte 24 Euro. *He had 24 euros.* Er freut sich über **das Verdienen** von 51 Euro. *He is happy about****the earning****of 51 euros. (glossing)* *He is happy about earning 51 euros. (translation)* Wie viel Geld hat der Mann jetzt? *How much money does the man have now?*
C14	Ein Mann spart Geld für einige Anschaffungen. *A man saves money on some purchases.* Er hatte 82 Euro. *He had 82 euros.* Er verdient 15 Euro. *He earns 15 euros.* Wie viel Geld hat der Mann jetzt? *How much money does the man have now?*
C15	Ein Mann spart Geld für einige Anschaffungen. *A man saves money on some purchases.* Er bedauert ***das Ausgeben*** von 52 Euro. *He regrets****the spending****of 52 euros. (glossing)* *He regrets spending 52 euros. (translation)* Er hat jetzt 24 Euro. *He has 24 euros now.* Wie viel Geld hatte der Mann. *How much money did the man have?*
C16	Ein Mann spart Geld für einige Anschaffungen. *A man saves money on some purchases.* Er hat 74 Euro ausgegeben. *He spent 74 euros.* Er hat jetzt 23 Euro. *He has 23 euros now.* Wie viel Geld hatte der Mann? *How much money did the man have?*

The sentences shown in italics provide English translations of the German examples used in the experiment. For the sentences with the nominal form, both a word-by-word glossing and a translation are provided to clearly indicate both the linguistic characteristics of the German example and its meaning. C1–C16 corresponds to the factors in this table

The 320 word problems contained ten different templates (see Table 3). Each template was structured in the same format and included four sentences. Each template contained all the factors. The order of problems was systematically varied to avoid ordering effects.

**Table 3:** Templates

	Templates
1	Eine Marktfrau verkauft Äpfel auf dem Markt *A market woman sells apples in the marketplace*
2	Ein Mädchen hat ihren Freunden Bücher mitgebracht *A girl brought books to her friends*
3	Einige Leute wurden zur Party eingeladen *Some people were invited to the party*
4	Ein Vater spielt mit Kindern beim Geburtstag Versteckspiel *A father plays with children at the birthday hide*-*and*-*play game*
5	Eine Studentin muss Wörter lernen *A student must learn words*
6	Ein Mann spart Geld für einige Anschaffungen *A man saves money on some purchases*
7	Ein Dieb hat einer Frau einige Diamanten gestohlen *A thief stole some diamonds from a woman*
8	Eine Tennisspielerin spielt auf der Weltprofitour Turniere im Einzel *A tennis player plays in the tournaments in single*
9	Peter möchte ein gebrauchtes Fahrrad kaufen *Peter would like to buy a second*-*hand bike*
10	Ein paar Freunde suchen Pilze im Wald *A few friends are looking for mushrooms in the forest*

Each word problem consisted of four sentences, which were presented simultaneously (for examples, see Table 2). The second and third sentences contained the two numbers and cue words necessary for the operation. The final fourth sentence contained the question.

The arithmetic operation was limited to addition or subtraction. All problems consisted of two-digit numbers in Arabic notation and overall problem size, as the number of various strategies increases with problem size (Verschaffel et al. 1998), was matched between non-carry and carry addition, as well as between subtraction and addition, problems (problem size: subtraction *M* = 76.7, SD = 15.1; addition: *M* = 82.5, SD = 18.8). Number pairs for the tasks were matched in difficulty because calculations with smaller numbers have been shown to be retrieved from a network of mental representations, while calculations with larger numbers require a transformation process (LeFevre et al. 1996). Therefore, because it has been shown that response times on simple arithmetic problems are in general slower and more error prone if the operands and their correct solutions are larger, we have controlled the problem size in our experiments (Klein et al. 2009).

Number pairs with identical numbers, “shot numbers,” with a zero in the units digit, mirror numbers (e.g., 24–42), and all combinations of the operand with the same numbers in the tens or units digits (e.g., tens: 41 + 43, units: 24–14) were excluded to prevent any automatic mental retrieval. The order in which the number size was presented in the task was balanced across all passes in equal proportions: Half of the stimuli started with the larger number and the other half with the smaller number, because although 4 + 2 = 6 and 2 + 4 = 6 are arithmetically equivalent, the processing may differ (Kaput 1979). For instance, Nys (2010) found that test subjects preferred when the larger number occurred first in mental addition. The number of tasks with even or odd results was balanced as well because parity also influences the difficulty of simple addition and subtraction (Hines 2013).

Lexical consistency or inconsistency was encoded through an operation lexically evoked in the text. The second or the third sentence contained a cue word (in verbal or nominal form), which evoked an operation (e.g., “sell”/“selling” for subtraction). Depending on the scenario of the story, the cue word could be misleading. For example, the German verb “leihen” can mean “to lend” or “to borrow,” which from the point of view of the lender evokes subtraction but from that of the borrower evokes addition. To make the perspective explicit, a reference introductory sentence was included to provide context before every task.

The type of nominalization in the text was formulated in the so-called infinitive-based nominalization. Generally, in nominalization the words lose their verbal characteristics and behave like real nouns (Hamm and van Lambalgen 2002). In German, there are two possibilities for creating nominal forms: one using the “–ung” suffix (e.g., “landen” “to land” becomes “Landung” “landing”) and the other based on the infinitive (“landen” becomes “das Landen”). The latter is very close in meaning to the underlying verbs, denoting the events or states that the verbs denote (Scheffler 2005). They can be formed for any German verb, whereas “–ung” nominalization is not available to all verbs. For preparing the stimuli, it was important that the nominalized form be applicable to all selected keywords (in our case verbs), and that the meaning stayed as close to the original meaning as possible, so we systematically used infinitive-based nominalization instead of “–ung” nominalization. All sentences were constructed with active verb forms given that active voice has been shown to facilitate word problem understanding (Abedi et al. 2005), and we wanted to avoid additional linguistic complexity. Compared to other studies (e.g., Prediger et al. 2015), which changed several noun phrases at once in the text, we kept the number of changed noun phrases as small as possible. For example, Spanos et al. (1988) showed that certain grammatical features (e.g., prepositional phrases, noun phrases) prevented participants from fully understanding mathematical word problems. In our study, the nominalized and verbal forms differ only as much as is necessary to realize the variants: The number of noun phrases differs because in one case a verb is used (e.g., verkauft), whereas in the other that verb is nominalized (e.g., den Verkauf) and embedded under another verb. Therefore, the sentence with the nominal form is syntactically more complex. Other conditions do not differ in syntactic complexity.

To minimize the risk that response time would be adversely affected by prolonged reading and the extra burden of working memory (Shaftel et al. 2006), filler words and information not relevant to understanding (Muth 1992) and solving the task were avoided. We kept the length, word count, and frequency as similar as possible given that lexical complexity (word frequency) and syntactic complexity [as reflected by the mean sentence length in words, item length in words, noun phrase length, and number of prepositional phrases (Abedi et al. 2005)] influence reading. The overall number of words and average sentence length are related to reading difficulty with longer sentences posing greater challenges for readers (Butler et al. 2004). Item length shows relatively consistent negative effects, increasing item difficulty (Abedi et al. 1997). Table 4 provides the average word count, character length, syllable count, character count per word, and syllable count per word for each condition as well as frequency measures. Frequencies were calculated using the COW German Corpus (Schäfer 2015; Schäfer and Bildhauer 2012).

**Table 4:** Linguistics measures across conditions

	Word count		Total character length		Total syllable count		F_logpermil_10		Band	
	Mean	SD	Mean	SD	Mean	SD	Mean	SD	Mean	SD
Addition										
Non-carry/non-borrow										
Consistent form										
Verbal form	26.15	3.47	127.85	24.14	41.15	8.74	128.14	10.58	7.85	0.53
Nominal form	30.00	3.42	145.55	22.91	46.90	8.19	130.9	11.54	7.70	0.53
Inconsistent form										
Verbal form	25.15	2.60	128.9	20.77	40.85	7.01	123.42	8.72	8.13	0.44
Nominal form	28.5	3.14	145.4	22.18	45.65	7.56	129.62	7.30	7.99	0.25
Carry/borrow										
Consistent form										
Verbal form	26.15	3.47	127.85	24.14	41.20	8.70	128.27	10.57	7.84	0.53
Nominal form	30	3.42	145.55	22.91	46.90	8.19	130.90	11.54	7.70	0.53
Inconsistent form										
Verbal form	25.15	2.60	128.90	20.77	40.85	7.01	123.42	8.72	8.13	0.44
Nominal form	28.45	3.15	145.40	22.18	45.65	7.56	129.62	7.30	7.99	0.25
Subtraction										
Non-carry/non-borrow										
Consistent form										
Verbal form	25.45	1.57	128.95	15.37	40.25	4.18	124.77	10.43	8.10	0.46
Nominal form	29.15	2.87	146.35	18.05	45.50	5.76	131.74	7.86	7.95	0.28
Inconsistent form										
Verbal form	26.45	2.78	130.90	19.74	41.70	7.31	127.36	10.73	7.90	0.58
Nominal form	30.25	3.13	147.30	18.67	46.75	6.45	130.55	11.04	7.77	0.54
Carry/borrow										
Consistent form										
Verbal form	25.45	1.57	128.95	15.37	40.25	4.18	124.77	10.43	8.10	0.46
Nominal form	29.15	2.87	146.35	18.05	45.50	5.76	131.74	7.86	7.95	0.28
Inconsistent form										
Verbal form	26.45	2.78	130.90	19.74	41.70	7.31	127.36	10.73	7.90	0.58
Nominal form	30.3	3.08	147.45	18.45	46.80	6.38	130.73	10.89	7.76	0.53

Word count, total character length, total syllable count, F_logpermil_10 (the log10 of the frequency per million shifted into the positive range by adding 10), band (frequency band)

Additionally, reading speed, reading comprehension, calculation for subtraction and addition, verbal working memory, visual working memory, and central executive were assessed for every individual. Because of length restrictions and because the N was too low in this study to derive strong conclusions about individual differences from multiple correlations, these results are only reported in the Supplementary Material 1 for the interested reader, but not discussed in the article itself. Generally, the correlations were in the expected direction; i.e., better reading and calculations skills were related to better performance on the word problems.

### Procedure

The experiment took place in a laboratory at the University of Tübingen. The stimulus was presented on a 19-inch monitor in white Times New Roman font, size 24, bold on a black background. Voice or spoken response was used to detect response time. For this purpose, 32 two-digit numbers were presented on the screen, divided into two blocks. For this, the Voice Key (Creative EMU 0202, USB audio interface) was used as a trigger, consisting of the interface and a headset with microphone. To reduce potential measurement inaccuracy (Kessler et al. 2002) or at least keep it constant, all participants were asked to loudly and clearly say, “is,” followed by the displayed number (e.g., in German “ist 45”). Subjects were instructed that only mental calculations were allowed and that no other calculation support (such as finger movements) should be used.

The 320 tasks were divided into two blocks, 160 tasks each, with a break in-between. Each task was presented separately in pseudo-randomized order. Presentation was adjusted so that in each block all 16 conditions were included. In addition, they were mixed, and a single condition could be presented no more than three times in a row. Participants in the experiment were seated such that the distance between their eyes and the monitor, on which the word problems were displayed, was approximately 70 cm. All four sentences were presented at the same time on the screen, aligned in the centered position, with one line containing one sentence. The stimuli were present until the Voice Key was triggered by the “Is(t)” cue. The response time was taken from the point that the stimuli was presented till the Voice Key was triggered. After the trigger, the participants saw a black background and communicated the solution. The investigator noted the response and other incidents during the experiment. A fixation point before each trial was used (*x*/*y* coordinates: 112/384).

## Results

### Analysis

Statistical analysis of RTs and accuracy data was performed using the R-project statistical computing software (Team 2014). Response times were analyzed using linear mixed effects models (LMM), and accuracy data were analyzed as a binomial variable using generalized linear mixed effects models (GLMM) as implemented by the lme4 package (Bates et al. 2014). In all of the subsequently considered models, independent random intercepts are included for both participants and items. Random slope models turned out to be too complex for the current data set, because estimation algorithms did not converge. Each factor was dummy coded, with the easier condition (forming the reference category) labeled by 0, and the more difficult condition by 1 (subtraction operation, inconsistent lexical form, carry/borrow, nominalized form). This means that the intercept corresponds to the mean response time (mean accuracy, respectively) of an item, which is in the reference category for all of the factors. Models were built up stepwise in a hierarchical way, and statistical decisions are based on incremental likelihood ratio tests (LR Chisq in subsequent tables). Beyond the random effects, the null model (Model 0) included an intercept only, and no other fixed effects. Model 3 formed the full model including all interactions for both RTs and accuracy. Table 5 provides means and standard deviations for all factor level combinations for both response time and accuracy.

**Table 5:** Response time, accuracy mean, and standard deviation for all 16 factors

	Reaction time		Accuracy in %	
	*M*	SD	*M*	SD
Total	9.37	2.54	0.93	0.03
Addition				
Non-carry/non-borrow				
Consistent form				
Verbal form	8.31	2.22	0.95	0.08
Nominal form	8.76	2.79	0.95	0.05
Inconsistent form				
Verbal form	8.57	2.19	0.92	0.06
Nominal form	8.81	2.35	0.94	0.07
Carry/borrow				
Consistent form				
Verbal form	9.26	2.21	0.95	0.07
Nominal form	9.94	2.57	0.93	0.06
Inconsistent form				
Verbal form	9.55	2.77	0.92	0.07
Nominal form	9.66	3.00	0.89	0.10
Subtraction				
Non-carry/non-borrow				
Consistent form				
Verbal form	8.67	2.53	0.95	0.06
Nominal form	8.69	2.35	0.95	0.06
Inconsistent form				
Verbal form	9.09	2.69	0.93	0.05
Nominal form	9.55	2.51	0.92	0.08
Carry/borrow				
Consistent form				
Verbal form	10.07	3.12	0.88	0.10
Nominal form	10.10	3.21	0.93	0.05
Inconsistent form				
Verbal form	10.46	2.55	0.91	0.06
Nominal form	10.76	3.39	0.92	0.08

### Response time

Adding the main effects carry, operation, lexical consistency, and nominalization to Model 0 one by one showed that all of them improve model fit. Model 1 included all of these main effects and formed the basis for testing interactions, which again were tested in a stepwise manner. Model comparison (see Table 6) revealed that the most parsimonious model exhibiting a good fit to the data was Model 2, containing all main effects as well as the interaction operation * lexical consistency. Including any additional interactions did not improve fit.

**Table 6:** Comparison of the LMMs in response time analysis

	Effects	*df*	AIC	BIC	LR Chisq	Δ *df*	*P* value
Model 0	Intercept only	4	32,753	32,781			
Model 1	Main effects added	8	32,644	32,698	117.48	4	< 2.2e−16
Model 2	Operation * consist. interaction added	9	32,640	32,702	5.52	1	0.019
Model 3	Full model	19	32,654	32,783	6.60	10	0.763

**Fig. 1:**
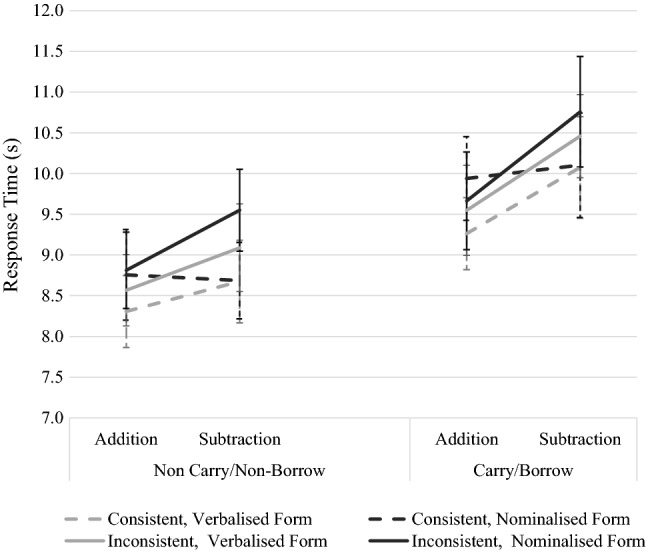
Response times separated for each factor. Error bars show standard error of the mean (SEM). Dark line is addition; gray is subtraction; solid line: carry/borrow, dashed line: non-carry/non-borrow factors

The estimates for Model 2 are listed in Table 8, together with standard errors and *t* values. Consistent with our first hypothesis, the following main effects turned out to be significant (see Fig. 1). Carry produced the most pronounced effect, increasing latencies by 1.16 s compared to problems without carry. Subtractions were only slightly slower than additions on average (0.30 s), and nominalization provided about the same effect size (0.28 s).

**Fig. 2:**
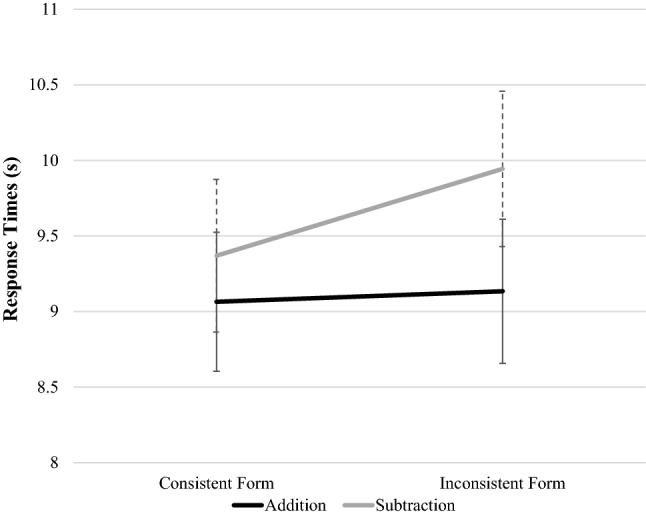
Two-way interaction between factors operation * lexical consistency. Error bars show standard error of the mean (SEM)

The two-way interaction (see Fig. 2) between the arithmetic factors operation and lexical consistency (0.54 s) indicated that there was a consistency effect (difference between lexically inconsistent form vs. lexically consistent form) for subtraction problems only (RTs increase by 0.60 s on average). For addition problems, lexical consistency had no effect (0.06 s). Random effects indicate that there was substantial individual variation in mean RT across participants (SD = 2.38), but items seemed to be quite homogenous (SD = 0.84).

In sum, the current results suggest that all factors wielded influence on the latencies, which for carry, operation, and nominalization can be described as main effects. Consistency effects were present for subtractions only.

### Accuracy

Comparing GLMM models in the same way as the LMM models for RTs reveals (see Table 7) that the model that describes the data best is Model 1. Its estimates are collected in Table 8. Model 1 includes fixed main effects for carry (log odds − 0.36) and lexical consistency (log odds − 0.29) only, and both are negative and thus are decreasing accuracy. Adding the other main effects (Model 2), or any interaction (Model 3) did not improve model fit. However, care should be taken in interpreting the accuracy results, due to the overall high accuracy (92%) indicating a ceiling effect (log odds 3.14 for the intercept). This is also reflected by the comparatively small individual variation captured by the random effects of participants (SD = 0.36) and items (SD = 0.65).

**Table 7:** Comparison of the GLMM models for accuracy

	Effects	*df*	AIC	BIC	LR Chisq	Δ *df*	*P* value
Model 0	Intercept only	3	3753.1	3773.8			
Model 1	Carry and consistency main effects added	5	3742.9	3777.4	14.24	2	< 0.001
Model 2	All main effects added	7	3746.3	3794.7	0.60	2	0.740
Model 3	Full model	18	3755.1	3879.5	13.18	11	0.282

**Table 8:** Model outputs for response time (Model 2) and accuracy (Model 1)

	Response time (s), Model 2			Accuracy (log odds), Model 1		
Random effects	Variance	SD		Variance	SD	
Item	0.71	0.84		0.42	0.65	
Subject	5.67	2.38		0.13	0.36	
Residual	6.69	2.59				

Fixed effects	Estimate	SE	*t*	Estimate	SE	*Z*
Intercept	8.38	0.5	16.9	3.14	0.13	23.24
Operation	0.30	0.16	1.86			
Lexical consistency	0.06	0.16	0.36	− 0.29	0.12	− 2.44
Carry/borrow	1.16	0.11	10.27	− 0.36	0.12	− 3.05
Nominalization	0.28	0.11	2.50			
Operation * lexical consistency	0.54	0.23	2.36			

Table 8 presents the model outputs for the final models for RT (Model 2) and accuracy (Model 1).

## Discussion

The main goal of this study was to explore key linguistic and arithmetic factors that may contribute to the difficulty many people experience when solving word problems. We manipulated linguistic complexity to be independent and orthogonal to the arithmetic complexity of the underlying word problem in such a way that there was one factor related to arithmetic/linguistic complexity and one that was unrelated. For example, the linguistic factor lexical consistency and the arithmetic factor operation were related to arithmetic complexity, whereas the linguistic factor nominalization and the arithmetic factor carry/borrowing were not. We examined the main effects and interactions of these manipulations on word problem performance and their connection with the problem solving process.

In our first hypothesis, we expected that all arithmetic and linguistic factors would have a main effect on performance (i.e., response time). The factors carry, operation, and nominalization all had a main effect on response time. Confirming the H1.1 hypothesis, it took participants more time to solve subtraction tasks as compared to addition tasks. Additionally, the main effect of carry indicated that participants had a greater response time when solving word problems with a carry/borrow operation as compared to tasks without this operation, which is in line with the H1.2 hypothesis. This is consistent with and extends similar findings in which arithmetic calculations were not embedded in a word problem (Geary et al. 2012; Imbo et al. 2007). This was also expected because the carry effect in addition tasks and the borrow effect in two-digit subtraction tasks are both associated with increased arithmetic difficulty (Deschuyteneer et al. 2005). The H1.3 hypothesis that word problems with an inconsistent form should take significantly longer to answer than those that are consistent was partially confirmed, because the consistency effect was present only in the word problems with subtraction. Here, our findings are consistent with the results of Hegarty et al. (1995) and Van der Schoot et al. (2009), and opposed to those of Verschaffel et al. (1992), who found a consistency effect in children but not in adults. Finally, the nominal form significantly increased response time compared to the verbal form, which confirms the H1.4 hypothesis. This result extends previous findings that nominalizations can be a source of difficulty (Abedi et al. 2005; Prediger et al. 2015). Contrary to studies that concluded that difficulty arises only in complex multi-step word problems with a high density of nominalization (Schlager et al. 2017), our study shows that simple nominalization influences the complexity of the word problems as well. Considering that the number of sentences, words, and even characters in this study were kept consistent between word problems, this finding provides support for the importance of unrelated linguistic factors. Generally speaking, the main effects support the direct influence of stimulus attributes on word problem performance as in the theoretical process model proposed by Daroczy et al. (2015).

### Interaction of linguistic and arithmetic factors

In the same model (Daroczy et al. 2015), however, we hypothesized that word problem difficulty comprises not only the linguistic complexity of the text and arithmetic complexity of the arithmetic problems but also the interaction of these factors because some attributes are processed at common stages. Therefore, we expected the interactions between arithmetic and linguistic factors to be more pronounced for the related factors than for the unrelated factors. As expected in the H2.1 hypothesis, operation interacted with lexical consistency. The interactions were particularly pronounced for factors relating to both text and arithmetic operation. There was a consistency effect for subtraction but not in the case of addition, suggesting an over-additive effect in the most difficult condition. The direction of this interaction between operation and lexical consistency is supported by some studies but not by others. For example, in this study the direction of the interaction was the opposite to that previously found by Verschaffel et al. (1992), where the difficulty of overcoming inconsistent language was enhanced in the case of addition and multiplication. An explanation as to why our results are different could be that the previous study used compare problems, which have a different semantic background from the change problems used in the current study. Although the interaction between lexical consistency and operation is quite stable across various studies, in accuracy there was no interaction of these factors, and it is important to note that the overall error rate was very low, which might indicate a ceiling effect. A possible explanation for this over-additive interaction is given by the model of Daroczy et al. (2015). Joint domain-general stages of processing like working memory have limited resources. Difficulty in both the linguistic and the arithmetic domain leads to particularly slow processing, perhaps because there may not be enough resources. For this interaction between operation and lexical consistency, however, another alternative interpretation is possible, using linguistic markedness. An interaction between markedness and lexical consistency in these studies is reflected in the fact that problem solvers find it especially difficult to solve a problem in the inconsistent-marked condition, i.e., inconsistent addition problems seem to be harder than inconsistent subtraction problems (Hegarty et al. 1995; Verschaffel et al. 1992). This interaction between operation and lexical consistency is explained by the fact that increased difficulty does not depend on the operation but on the greater semantic complexity of the marked sentences. Nevertheless, the problem with this interpretation is—unlike the factor nominalization in this study—markedness cannot be manipulated independently from lexical consistency and operation (Van der Schoot et al. 2009). Namely, consistent and marked-inconsistent problems always concern addition, whereas the two other problem types (unmarked-inconsistent and marked-consistent) always concern subtraction, so it is still unclear whether the semantic change or the interaction of operation and language causes this difficulty. This needs to be disentangled in the future. Our findings suggest that the interaction between operation and lexical consistency does not only depend on the semantic and arithmetic features, but also the interaction of language and mathematics.

Although we have not found a significant interaction between nominalization and other factors in the linear mixed effect models, in the case of nominalization, the consistency effect for response times was slightly more pronounced for subtraction problems than for addition problems, something we did not observe in the verbal forms. Therefore, we suppose that in the linguistically less demanding conditions there are enough cognitive resources to process the complex lexical consistency and the complex operation condition in combination. However, when the text gets linguistically difficult (which requires additional resources), there are no resources left for this most demanding interaction condition, which leads to slower responses. This might especially hold for individuals with lower cognitive abilities or who are in an earlier developmental stage, children, for example.

Finally, in the case of carry, i.e., for the arithmetic factor, which was hypothesized to interact less with linguistic factors, because it was unrelated, there was no interaction with other linguistic factors. This partially supports the hypothesis that for factors affecting only arithmetic the interactions may be absent or less consistent (H2.2).

In sum, the interaction between arithmetic and linguistic factors speaks to possible joint sources of linguistic and arithmetic difficulty. Carry produced the most pronounced effect; subtractions were only slightly slower than additions on average and nominalization had about the same effect size on the response time compared to operation. The consistency effect was significant for subtraction problems only.

### Underlying cognitive processes in solving arithmetic problems

#### Problem solving models

The proposed models for how word problems are solved disagree on the origin of the internal problem model, i.e., whether the internal problem representation is a result of a schema or a situation—i.e., mental representation model. The findings of this study support the situation or mental representation model over the schema model (Thevenot and Barrouillet 2015; Thevenot and Oakhill 2005). Provided that in the schema model only the keyword matters and the difficulty of the inconsistent word problem results from the mismatch between text and schema (Kintsch and Greeno 1985), it may be suggested that in this study only nominalization and lexical consistency should result in significant differences (when only the keyword is affected) if this was the appropriate model. On the contrary, we found effects for all other factors, as well as an interaction. Additionally, the word problems with nominalized, inconsistent form and subtraction resulted in the highest response time, which can be interpreted as the additional text difficulty making it harder to create a problem model in this mathematically more difficult condition. In fact, the interaction also holds for the situation model that requires the construction of a mental representation of the situation described by the problem (Johnson-Laird 1983).

#### Interactions between related and unrelated factors and the problem solving models

Additionally, the problem solving models mentioned above did not agree on whether the problem solving phases are fully separable or not. Manipulating related and unrelated factors at the same time might provide elaboration on how the presence or absence of an interaction between the factors could support the existing models mentioned above. In particular whether or not the initial reading phase and the last calculation phase interact with other problem solving phases, like the mental representation for example, could be addressed in future studies.

*The relation of calculation phase to other phases of problem solving* In this study we have manipulated computation with two factors. In one the number difficulty (i.e., carry) was changed, and in the other, the difficulty of the operation was changed to gain a better understanding of whether the computational process affects the mental model or not. If computation also affects the problem model phase, where the mental model is built, we would have found an interaction between the unrelated arithmetic factor carry and the other factors. However, we found no interactions between carry and the other arithmetic and linguistic factors. These results are favored by propositional theory, which sees the calculation phase and the other problem solving phases as distinct. This finding is in line with Rabinowitz and Wooley (1995) who found no interaction for either response time or for accuracy between the factors problem size, carry, and others. This means that in this study we found no evidence that number difficulty would affect another stage of problem solving—i.e., the mental representation, besides the calculation phase. Therefore, in the case of carry, sequential processing is highly probable, and it likely affects the problem solving phase after the creation of the mental model and does not influence the quality of the representation. However, there might be an alternative interpretation. Failing to solve a word problem successfully is priory hypothesized due to the failing of the creation of the mental model (Hegarty et al. 1995; Verschaffel et al. 1992). Nevertheless, the factor carry affected the correctness of the solution. This means that it might be hard to determine from a non-correct solution if it is due to the (1) the correct mental representation and wrong calculation or (2) to the incorrect mental representation because, for example, the incorrect calculation can be primed by specific words (Bassok et al. 2008). A second alternative explanation would be that the carry operation also gets more difficult as textual processing gets more difficult, but the missing interaction with other factors is at odds with this explanation.

*The relation of reading phase to other phases of problem solving* The arithmetically unrelated linguistic factor (i.e., nominalization) that did not change the underlying mathematics did not show an interaction with other factors. However, an interaction between nominalization and the other factors would have suggested that the reading comprehension phrase interacts with other problem solving phases. The results again favor the propositional model over the non-sequential cyclic models. This is not exactly in line with the hypothesis derived from the model of Daroczy et al. (2015) which would expect an interaction also in case of factors where the cognitive load increases. The missing interaction between unrelated linguistic and arithmetic factors could also mean that it is not correct to assume a limited load domain-general stage model as described in the model of Daroczy et al. (2015). Nevertheless, nominalization showed a main effect on response time that confirms the direct influence route from the model. Therefore, a possible interpretation is that the arithmetically unrelated linguistic factor (i.e., nominalization) might affect only the initial reading phase. The accuracy results support this, as there was no significant main effect for nominalization, nor an interaction with other factors. On the other hand, the interaction between lexical consistency and operation might mean that in the case of lexical consistency the process of reading is not completely separable from the process of solving. However, this interaction between lexical consistency and operation is consistent with both the sequential and cyclic models.

In summary, we can say that the absence of an interaction between the unrelated and related factors is more consistent with the propositional theory than with the cyclic model. This might imply that the initial reading phase, the calculation phase, and the building of the problem model phase can be viewed as distinct, non-overlapping stages of problem solving. As no interaction was found between carry and nominalization or between other factors, these factors might influence different processing stages—cf., Sternberg (1969). Number difficulty (i.e., carry) seemed to affect the calculation/execution phase, and text difficulty (i.e., nominalization) the initial text comprehension phase.

### Theoretical implications

Mathematical texts have their own terminology and language, and it is claimed that they require special literacy skills that are developed across years (Burton and Morgan 2000). Additionally, it is suggested that word problems and calculations represent distinct domains of mathematical performance (Fuchs et al. 2008; Swanson 2004). Language is assumed to have a stronger effect on word problem solution success than, for example, calculation skills (e.g., Fuchs et al. 2014). This hypothesis is supported by the documented strong connection between text comprehension and word problem solving (Boonen et al. 2013, 2014; Kintsch and Greeno 1985; Vilenius-Tuohimaa et al. 2008) and by the observation that some students who have otherwise little to no issues with arithmetic tasks cannot solve those that are written in textual form (Nesher and Teubal 1975). Because of this evidence, word problem research and intervention often focuses mainly on text difficulty and the problem of constructing an adequate representation (Cummins et al. 1988; Davis-Dorsey et al. 1991; De Corte et al. 1985; Lewis and Mayer 1987). Therefore, recent studies suggest a stronger focus should be placed on training mathematical literacy and reading skills (e.g., Fuchs et al. 2008). In our study, we observed interactions between arithmetic factors and linguistic factors but also that arithmetic difficulty plays a strong role on its own. Therefore, we suggest it might be an incomplete approach to mainly focus on text difficulty in word problems. Instead, mathematical and language requirements need to be considered together when designing word problems. Therefore, in addition to better text comprehension instruction when teaching word problem solving strategies, we suggest that the involvement of arithmetic factors should not be neglected, even if children are able to solve equivalent mathematical calculations in less resource-demanding tasks. Instruction in text comprehension or mathematics may even need to be individually adapted. In some types of word problems, arithmetic difficulty might play a role, while in others linguistic difficulty is more important. Our suggestion is to confront children with a particular type of word problem, namely the problem from which they can learn most.

### Limitations of the study and further perspectives

Integral to this study is the systematic investigation of select linguistic and arithmetic factors to distinguish from where the difficulty of word problems originates. For example, when faced with two word problems with the same arithmetic operations but different textual difficulties, an individual may not be able to create a problem representation for the textually more difficult problem. Another outcome might be that despite the correct problem representation and otherwise good mathematic skills, a calculation error occurs due to the increased cognitive load from the interaction between arithmetic and linguistic factors. In both cases the result is the same, namely an incorrect solution. However, each case would require a different intervention. Therefore, we suggest that further elements of text characteristics that cause difficulty for various individuals should be systematically investigated (see Supplementary Material). Other arithmetic and linguistic factors that are potential candidates for systematic study include number sense (Dehaene 2001), the presented order of the numbers, and the influence of these features on solution accuracy, paired with cognitive ability and item difficulty. For instance, this study did not include factors that did not change the underlying structure but manipulated the calculation process. For example, Bassok et al. (1998) have shown that calculations represented by functionally connected words are easier to add up. Such a linguistic factor could be a candidate to show an interaction between the calculation phase and the reading phase. This is supported by Bagnoud et al. (2018) who found that brain activity differs when exposed to discrete quantities or continuous quantities (e.g., apples, meter, rope).

It is important to note that the current paper did not cover and investigate all the elements of the proposed theoretical word problem solving process model from Daroczy et al. (2015) because the model also suggests a connection not only to task characteristics but to individual abilities or to individual strategies, as well as the inclusion of the role of the environment. The current study was conducted in adults to examine the factors that influence word problem difficulty. However, selecting participants from other groups might lead to different results. For instance, examining a group of children, who, when compared to adults, have a more limited working memory capacity and therefore experience higher cognitive load (Swanson and Beebe-Frankenberger 2004) might lead to more over additivity than that observed in adults. Finally, it is important to note that the word problem is a didactical construct which serves purposes in mathematical education from simple exercises on basic operations (Greer 1997) to more complex tasks (Verschaffel et al. 1997). Performance when solving word problems is highly influenced by the environment and what is expected in the learning scenario (Cobb and Bauersfeld 1995). Therefore, in future diagnostic assessment both the item characteristics (i.e., the linguistic and arithmetic complexity) and the individual characteristics (i.e., general linguistic and mathematical skills), as well as the environment, should be considered.

## Conclusion

In the current study, we investigated the influence of arithmetic and linguistic factors on word problem performance. We argue that it is essential to investigate the influence of arithmetic and linguistic factors on word problem performance because: (1) the connection between mathematical and linguistic factors gives rise to the difficulty of word problems, and (2) one needs to distinguish factors where linguistic and mathematical aspects are conceptually linked (e.g., consistency of verb meaning with the mathematical operation to be performed) from linguistic and arithmetic factors that are not linked in the other domain (e.g., nominalization, carry) to identify if or when interaction effects are to be expected. Our results indicate that both linguistic and arithmetic complexity contribute to the difficulty of a word problem and that linguistic and arithmetic factors interact. Therefore, linguistic and arithmetic complexities are not always fully separable attributes.

## Electronic supplementary material

Below is the link to the electronic supplementary material.

Supplementary material 1 (DOCX 39 kb)
