# Supplementary material for: The interaction of linguistic and arithmetic factors affects adult performance on arithmetic word problems
Source: Cogn Process. 2020 Jan 22;21(1):105–25. doi: 10.1007/s10339-019-00948-5 (PMC7002335; doi:10.1007/s10339-019-00948-5)
Supplement: Supplementary file 1 — Supplementary material 1 (DOCX 39 kb) [file 10339_2019_948_MOESM1_ESM.docx]

Supplementary Material for the Article:

The Interaction of Linguistic and Arithmetic Factors Affects Adults’ Performance on Arithmetic Word Problems

**Characteristics of the Individual**

In the main article, we have been concerned with the characteristics of word problems, i.e. their linguistic and arithmetic complexity. However, individual cognitive abilities such as reading skills, working memory, mathematical ability and other skills play an important role in word problem success. It is still an open question which above mentioned components play a role in different types of word problems. For instance, linguistic capabilities could be more important for linguistically complex word problems, while arithmetic capabilities could be more important for arithmetically complex word problems. Due to the relatively low number of participants, our study is not conclusive here, but it already gives some hints about individual differences, which might be important in word problems. Therefore, we would like to give this information to the interested reader but will not draw strong claims from these data.

The following individual characteristics were studied for the following reasons:

*Working memory.* It has been suggested that word problem performance in general is strongly related to working memory and a strong relationship has been documented between individual differences in WM and performance in arithmetic word problems (Adams & Hitch, 1997; Passolunghi & Siegel, 2001; Swanson, 2004; Swanson, Cooney, & Brock, 1993). Word problem solving is hypothesized to show a stronger correlation with working memory than basic number knowledge (Peng, Namkung, Barnes, & Sun, 2016). Although mathematical skills significantly affect the relationship between working memory and mathematics and for example Imbo, Vandierendonck, and De Rammelaere (2007) emphasized the role of working memory in mental arithmetic tasks, a number of studies have also shown that the correlation between working memory and problem solving accuracy is substantially lower when corrected for differences in reading comprehension (Fuchs et al., 2006).

*Reading and mathematical skills.* Successful word problem solving may also be related in part to basic reading and mathematical skills instead of any individual differences in working memory components (Swanson, Lussier, & Orosco, 2015; Zheng, Swanson, & Marcoulides, 2011). Word problems are introduced in schools after children have learned the formal operations of addition and subtraction (De Corte & Verschaffel, 1987), so many papers argue for the importance of reading skills in word problem solving as opposed to arithmetic problem solving skills. For instance, Fuchs et al. (2006) argue for reading skills alone, as mediator variables between working memory and word problem solving (but see Passolunghi and Siegel (2001) for opposite suggestions).

The mechanisms and implications of both skills are still under debate. On the one hand, reading comprehension is hypothesized to have an indirect effect on word problem solving performance via its influence on relational processing (Lee, Ng, & Ng, 2009). On the other hand, studies by Tolar et al. (2012) and Swanson (2006) indicate that processing speed plays a unique role in arithmetic, whereas language comprehension uniquely predicts accuracy of word problem solutions. Therefore, participants with better reading skills should be less affected by linguistic complexities. Although reading ability is positively related to scores on tests of arithmetic problem solving (Aiken Jr, 1971), children who have difficulties reading do not always perform poorly in mathematics. At the same time, arithmetic abilities are not often referred to or measured (Boonen, van der Schoot, van Wesel, de Vries, & Jolles, 2013). A task that is more demanding in arithmetic processing is also more demanding in the whole solution process of the word problem. Therefore, participants with better mathematic skills should be less affected by arithmetic complexity.

**Assessments**

To examine whether characteristics of the individual, and not only characteristics of the problems, influence word problem solving we assessed reading comprehension and reading speed, mathematical abilities (Fuchs, Fuchs, Compton, Hamlett, & Wang, 2015), and working memory. Our hypothesis is that subjects with higher working memory scores, better arithmetic abilities and better language comprehension skills will solve problems faster. We also suggest that individuals with better arithmetic abilities are less affected by arithmetic item complexity and individuals with better linguistic skills are less affected by linguistic item complexity.

*Working memory assessment.* Two tests provided working memory measures. Verbal working memory performance was tested with the letter span back and forward tests. To test the spatial memory span, a Corsi block-tapping test (Corsi, 1972) was used. After three consecutive errors the test was aborted. See Table 1 for the descriptive measures.

*Reading skill assessment.* To measure reading abilities, we used a *speed of reading- and reading comprehension test*, which was designed for grades six through 12 (Schneider, Schlagmüller, & Ennemoser, 2007). This test is a power test (so it was considered adequate to measure adults) and measures the speed of reading as well as reading comprehension.

*Mathematical skill assessment.* To measure basic mathematical skills, we used two short, self-made speed calculation tests for addition and subtraction (Huber, Fischer, Moeller, & Nuerk, 2013). Both tests contained 28 math problems and no text. All problems consisted of two-digit numbers (the pairs of numbers were different from those used for the word problems). Participants had 90 seconds to solve each sheet. As an independent variable, we used the number of correctly solved items.

-----------------------------------

Insert Table 1 about here

-----------------------------------

**Results on the influence of individual capabilities on word problem performance**

**Correlations and regressions for overall performance**

To examine other mechanisms that may have affected word problem solving, correlations of the individual cognitive abilities with the overall mean of the response time were calculated (see Table 2 for a table of the raw correlations between the included variables). The following individual cognitive abilities were included in the analysis: speed calculation tests in addition and subtraction (i.e., the number of correctly solved tasks in 90 s), reading comprehension (points achieved, resulting from the number of correctly identified words), reading speed (number of words read in 4 minutes), working memory (verbal: letters forward, spatial: cube episode forward, central executive: mean value of letter span and Corsi block sequence backwards). We conducted several multiple linear stepwise regressions: Response latency can be negatively predicted from reading comprehension and speed calculation (addition) [R^2^ = . 775, adjusted R^2^ = .754, *F*(2, 22) = 37.87, *p* <.000]. The correlation yielded better reading comprehension and mathematical skills result in lower reaction times.

-----------------------------------

Insert Table 2 about here

-----------------------------------

**Effect approach analysis predictions from specific competencies on overall performance on word problems**

We predicted that item characteristics relate to individual characteristics and that individuals with higher abilities in one domain were less affected by more complexity in that domain. To test this hypothesis, we have computed multiple step regressions of different individual capabilities for the response time on the consistency effect, on the nominalization effect, the carry effect, and the operation. Measures of individual capabilities were calculation skills, reading speed, reading comprehension (verbal as well as letters-span) and Corsi block backwards and forwards; these were included in the regression analyses as predictors.

For the **consistency effect** the regression model [R= .69, adjusted R^2^= .22, F(1, 23) =1.87,p=.137] turned out not to be significant.

For the **nominalisation effect** the final regression model [R= .44, adjusted R^2^= .16, F(2, 22) = 10.42, p=.001] included the following predictors: (i) reading comprehension, (ii) Corsi block forward test. The positive beta indicates that a relatively higher score in the reading comprehension (β=0.471) task was associated with a lower nominalisation effect, and the negative beta score of the Corsi block forwards (β=-0.421) indicated that a higher score was associated with higher nominalisation effect.

For the **operation effect** the final regression model [R= .57, adjusted R^2^= .292, F(1, 23) =10.89,p=.003] included only the predictor, speed test subtraction. The positive beta (β=0.567) indicates that a relatively higher score in the subtraction speed calculation task was associated with a lower operation effect.

For the **carry effect** the final regression model [R= .51, adjusted R^2^= .26, F(1, 23) =8.04,p=.009] included only the predictor, speed test subtraction. The positive beta (β=0.509) indicates that a relatively higher score in the subtraction speed calculation task was associated with a lower carry effect.

**Discussion of the influence of individual capabilities**

To examine whether characteristics of the individual also influence word problem solving we assessed reading comprehension, reading speed, mathematical abilities and working memory. The correlations in the Table 2 seem to hint that people with higher reading and mathematical abilities perform better when solving word problems: Numeracy and literacy skills had a significant negative correlation with reaction time and turned out to be significant predictors in the regression as well. However, surprisingly, individual working memory capability had no connection to reaction time in our study. This is unusual because many past studies (Adams & Hitch, 1997; Furst & Hitch, 2000; Passolunghi & Siegel, 2001; Swanson, 2004) have shown a strong influence of working memory on mathematical performance, and only a few studies did not find working memory to be a significant predictor (Fuchs et al., 2015). There are at least five reasons for the surprising finding that working memory did not predict word problem performance. First, the method of presentation might also increase the working memory demand. In the present study, the word problems were present until the answer was submitted, and participants could reread the problems when desired. Second, it is possible that we have not seen an effect for working memory in adults because it may play a different role, e.g. the extra information should be less distracting than for children. Third, most studies have been conducted in children, while we tested adults. Children vary more widely in reading and other cognitive skills than adults (Kingsdorf, Krawec, & Gritter, 2016). Thus, the divergence in findings may be just a matter of working memory variance within the tested sample. Fourth, different assessments of working memory may lead to divergent results as different measures of working memory are often poorly correlated. Fifth, in the current study we have used one-step word problems, which might have led to lower cognitive load. According to Peng et al. (2016), multistep mathematical tasks that require the calculation and maintenance of intermediate values are hypothesized to draw more on working memory resources than mathematical tasks that consist of fewer steps. Thus, there are multiple potential reasons why individual working memory capability predicted performance in some studies, but not ours.

**Influence on arithmetic and linguistics effects**

The hypothesis that participants with better mathematical skills should be less affected by arithmetic complexity, and participants with better reading skills should be less affected by the linguistic complexities could be partially confirmed.

**Arithmetic Effects.** For both arithmetic effects, namely the operation effect (explained variance: 29%) and carry effect (explained variance: 26%) the final regression model included only the predictor, speed test subtraction. This suggested that a relatively higher score in the subtraction speed calculation task was associated with a lower effect size, i.e. more arithmetically more capable individuals had less difficulties with the more complex arithmetic conditions. Furthermore, the carry effect correlated positively with reading comprehension and reading speed. This is especially interesting because carry/borrow is an arithmetic factor unrelated to linguistics factors, yet we can still see correlations with reading comprehension and reading speed.

**Linguistics Effects**. In the case of the nominalization effect – linguistics factor unrelated to arithmetic – the final regression model (explained variance: 16%) included the predictor reading comprehension. Higher scores in reading comprehension lower the nominalisation effect, i.e., better readers had less problems with more complex grammatical structures. However, a high score in Corsi block forward was associated with a higher nominalisation effect. This result is surprising, but more working memory capacity can have disadvantages and lead individuals to employ complex strategies in problem solving that are less optimal for a given task (DeCaro & Wieth, 2016). For the consistency effect, no individual skill turned out to be a significant predictor.

**Limitations.** Again, we wish to note that the N was rather small for our correlational and regression-based analysis. Some results, and especially some null results, may be due to the low power of this study. Therefore, we report these results only in this SOM, but not in the main paper. Although they generally correspond to the a priori hypothesis, they should be interpreted with care.

**Summary**

Individual reading and arithmetic abilities, but not working memory capacity, predicted overall performance. In effect-based analyses, in general, individual arithmetic capabilities predicted the arithmetic effects and individual linguistic and working memory capabilities predicted one, but not the other linguistic effect. Therefore, the data are consistent with the idea that arithmetically complex word problems are particularly difficult for students with poor arithmetic capabilities and linguistically complex word problems are particularly difficult for students with poor reading skills. With all due caution because of the low N, the data suggest that it could be promising to tailor the different arithmetic and linguistic complexities of word problems to the individual arithmetic and literacy capability of an individual

**References**

Adams, J. W., & Hitch, G. J. (1997). Working memory and children's mental addition. *Journal of experimental child psychology, 67*(1), 21-38.

Aiken Jr, L. R. (1971). Verbal factors and mathematics learning: A review of research. *Journal for Research in Mathematics Education*, 304-313.

Boonen, A. J., van der Schoot, M., van Wesel, F., de Vries, M. H., & Jolles, J. (2013). What underlies successful word problem solving? A path analysis in sixth grade students. *Contemporary Educational Psychology, 38*(3), 271-279.

Corsi, P. M. (1972). Human memory and the medial temporal region of the brain.

De Corte, E., & Verschaffel, L. (1987). The effect of semantic structure on first graders' strategies for solving addition and subtraction word problems. *Journal for Research in Mathematics Education*, 363-381.

DeCaro, M. S., & Wieth, M. B. (2016). When higher working memory capacity hinders insight. *Journal of Experimental Psychology: Learning, Memory, and Cognition, 42*(1), 39.

Fuchs, L. S., Fuchs, D., Compton, D. L., Hamlett, C. L., & Wang, A. Y. (2015). Is Word-Problem Solving a Form of Text Comprehension? *Scientific Studies of Reading, 19*(3), 204-223.

Fuchs, L. S., Fuchs, D., Compton, D. L., Powell, S. R., Seethaler, P. M., Capizzi, A. M., . . . Fletcher, J. M. (2006). The cognitive correlates of third-grade skill in arithmetic, algorithmic computation, and arithmetic word problems. *Journal of educational Psychology, 98*(1), 29.

Furst, A. J., & Hitch, G. (2000). Separate roles for executive and phonological components of working memory in mental arithmetic. *Mem Cognit, 28*(5), 774-782. Retrieved from http://www.ncbi.nlm.nih.gov/pubmed/10983451

Huber, S., Fischer, U., Moeller, K., & Nuerk, H.-C. (2013). On the interrelation of multiplication and division in secondary school children. *Frontiers in psychology, 4*.

Imbo, I., Vandierendonck, A., & De Rammelaere, S. (2007). The role of working memory in the carry operation of mental arithmetic: Number and value of the carry. *The quarterly journal of experimental psychology, 60*(5), 708-731.

Kingsdorf, S., Krawec, J., & Gritter, K. (2016). A broad look at the literature on math word problem-solving interventions for third graders. *Cogent Education, 3*(1), 1135770.

Lee, K., Ng, E. L., & Ng, S. F. (2009). The contributions of working memory and executive functioning to problem representation and solution generation in algebraic word problems. *Journal of educational Psychology, 101*(2), 373.

Passolunghi, M. C., & Siegel, L. S. (2001). Short-term memory, working memory, and inhibitory control in children with difficulties in arithmetic problem solving. *Journal of experimental child psychology, 80*(1), 44-57.

Peng, P., Namkung, J., Barnes, M., & Sun, C. (2016). A meta-analysis of mathematics and working memory: Moderating effects of working memory domain, type of mathematics skill, and sample characteristics. In: American Psychological Association.

Schneider, W., Schlagmüller, M., & Ennemoser, M. (2007). *LGVT 6-12: Lesegeschwindigkeits-und-verständnistest für die Klassen 6-12*: Hogrefe Göttingen.

Swanson, H. L. (2004). Working memory and phonological processing as predictors of children’s mathematical problem solving at different ages. *Memory & cognition, 32*(4), 648-661.

Swanson, H. L. (2006). Cross-sectional and incremental changes in working memory and mathematical problem solving. *Journal of educational Psychology, 98*(2), 265.

Swanson, H. L., Cooney, J. B., & Brock, S. (1993). The Influence of Working Memory and Classification Ability on Children′ s Word Problem Solution. *Journal of Experimental Child Psychology, 55*(3), 374-395.

Swanson, H. L., Lussier, C. M., & Orosco, M. J. (2015). Cognitive strategies, working memory, and growth in word problem solving in children with math difficulties. *Journal of learning disabilities, 48*(4), 339-358.

Tolar, T. D., Fuchs, L., Cirino, P. T., Fuchs, D., Hamlett, C. L., & Fletcher, J. M. (2012). Predicting development of mathematical word problem solving across the intermediate grades. *Journal of educational Psychology, 104*(4), 1083.

Zheng, X., Swanson, H. L., & Marcoulides, G. A. (2011). Working memory components as predictors of children’s mathematical word problem solving. *Journal of experimental child psychology, 110*(4), 481-498.

***Appendices***

Table 1

*Descriptive Measures (N=25)*

|  |  | Memory | | | | | | | | | | | | |  | | | | | | Mathematical and Reading Skill | | | | | | | | | | |
| --- | --- | --- | --- | --- | --- | --- | --- | --- | --- | --- | --- | --- | --- | --- | --- | --- | --- | --- | --- | --- | --- | --- | --- | --- | --- | --- | --- | --- | --- | --- | --- |
|  |  | Verbal Memory | | | | | | | |  | | Visual Memory | | | |  | |  | | | Mathematical Skill | | | |  | | Reading Measures | | | | |
|  | Age | Letter_Span Forward | | | Letter_Span Backward | |  | | Corsi_Block  Forward | | | Corsi_Block Backward | | Central Executive |  | | | | Addition | | | Subtraction | | | |  | | Reading Comprehension | | | Reading Speed |
| Mean | 22..08 | | 8.92 | 8.00 | |  | | 5.92 | | | 5.84 | | 6.92 | | | |  | | | 21.32 | | | 16.8 |  | | | | | 23.04 | 981 | |
| SD | 2.59 | | 1.92 | 1.77 | |  | | 1.09 | | | 0.97 | | 1.05 | | | |  | | | 4.55 | | | 3.71 |  | | | | | 9.20 | 329.22 | |

| Table 2  *Pearson Product- Moment Correlations of the cognitive variables and response latency (N=25)* | | | | | | | | | | | |  | |  | |  |  |
| --- | --- | --- | --- | --- | --- | --- | --- | --- | --- | --- | --- | --- | --- | --- | --- | --- | --- |
| Scale | 1 | 2 | 3 | 4 | 5 | 6 | 7 | 8 | 9 | 10 | 11 | | 12 | | 13 | | 14 |
| 1 Response Latency | - |  |  |  |  |  |  |  |  |  |  | |  | |  | |  |
| 2. Consistency Effect | -.19 | - |  |  |  |  |  |  |  |  |  | |  | |  | |  |
| 3. Nominalisation Effect | -.59** | .28 | - |  |  |  |  |  |  |  |  | |  | |  | |  |
| 4. Operation Effect | -.45* | -.01 | .52** | - |  |  |  |  |  |  |  | |  | |  | |  |
| 5. Carry Effect | -,47* | .06 | .45^*^ | .36 | - |  |  |  |  |  |  | |  | |  | |  |
| 6 Reading Comprehension | -.81** | -.35 | .56** | .19 | .50* | - |  |  |  |  |  | |  | |  | |  |
| 7 Reading Speed | -.77** | .20 | .51** | .20 | .50* | .96** | - |  |  |  |  | |  | |  | |  |
| 8 Corsi Block Forwards | .15 | -.15 | -.52** | -.16 | -.03 | -.22 | -.17 | - |  |  |  | |  | |  | |  |
| 9 Corsi Block Backwards | -.09 | -.02 | -.06 | .19 | ,14 | -.10 | -.15 | .42* | - |  |  | |  | |  | |  |
| 10 Letter Span Forward | -.01 | -.17 | .11 | .09 | ,01 | -.00 | .01 | .035 | .20 | - |  | |  | |  | |  |
| 11 Letter Span Backward | -.26 | .11 | .31 | .14 | -.00 | .35 | .33 | -.22 | -.04 | ,40* | - | |  | |  | |  |
| 12 Central Executive | -.28 | .09 | .25 | .22 | .065 | .26 | .23 | .00 | .44* | .45* | .88** | | - | |  | |  |
| 13 Speed Test Addition | -.71** | -.03 | .29 | .52** | .28 | .50* | .46* | .08 | .33 | .04 | -.06 | | .10 | |  | |  |
| 14 Speed Test Subtraction | -.60** | -.20 | .34 | .57** | .51** | .42* | .43* | .12 | .56** | .07 | -.01 | | .25 | | .65** | | - |
| MS | 9.37 | -2.65 | -2.29 | -4.54 | -9.37 | 23.04 | 981.00 | 5.76 | 5.76 | 8.92 | 7.76 | | 6.76 | | 21.32 | | 16.80 |
| SD | 2.42 | 2.86 | 2.93 | 3.83 | 6,33 | 9.39 | 336.01 | .93 | .93 | 1.96 | 1.74 | | .97 | | 4.64 | | 3.79 |

** p < .01. * p < .05
